# Persistence, Not Panic

**Published:** 1887-03-26

**Authors:** 


### PERSISTENCE, NOT PANIC.

Few deserve greater credit and more gratitude at the hands of the voluntary hospitals and their supporters than Sir Rutherford Alcock. For very many years he has laboured to promote the prosperity and to secure the efficiency of the London hospitals. In season and out of season, by his pen and on the platform, has Sir Rutherford advocated the claims of these institutions to increased and ever-increasing support. As Chairman of the Committee of more than one of the large hospitals, and as a member of many committees, he has worked long and successfully in this good cause. All must therefore gratefully recognise his services to the hospitals, and give favourable consideration to anything which he may advance concerning them.

In a long letter published in *The Times* of Saturday, the 19th instant, Sir Rutherford Alcock maintains that the pecuniary distress of the London hospitals to-day is overwhelmingly great; that, unless some extraordinary, not to say superhuman effort is made in their behalf, managers must face the facts and close their doors. Sir Rutherford's words are:—"It is obvious the inevitable result of the failure to obtain by voluntary contributions the larger support required from this source must be the total closure or partial suspension of the metropolitan institutions which succour and relieve the working classes when disabled by serious sickness or accident." This is undoubtedly true, unless, as he goes on to show, increased efforts are successfully made to raise the income of the hospitals to the requisite extent. To declare authoritatively that any leading metropolitan hospital is on the verge of financial ruin, and so within a measurable distance of dissolution, is to use the language of panic rather than the language of fact. No good, but the reverse, will come from any such panic-stricken utterances. The present position of affairs demands persistent advocacy alone, on the part of the managers of each London hospital, who must quit themselves like men. Then, but not till then, will all, and probably more than all the money which is required by the metropolitan hospitals be forthcoming.

Let us, therefore, change the language of panic to that of sober sense, and so try to discover how the present financial pressure may be successfully removed. We take it that everybody will agree that each London hospital requires the services of Governors with time and influence to devote to its interests as members of the governing committee. Many London hospitals have suffered almost necessarily from the absence of sustained and active interest in their welfare on the part of the large majority of those whose names appear upon the committees of management. There are exceptions, no doubt, to this rule; but there is reason to fear that if a statement could be prepared of the attendances of the members of every hospital committee of the metropolis, the absentees would far outnumber the workers. It matters not whether the governing body be a close borough like that of Guy's Hospital, or an elective and representative body like the committee of a voluntary hospital. In either case the drones should be eliminated from the hive, and workers substituted for them on the Committees of all the hospitals. Unfortunately, workers are not readily forthcoming for this duty in London, and so the remedy for the evil rests almost, if not quite entirely, with the Governors, that is, with the public who support these institutions.

Co-operation and combined effort is another force which should be brought to bear by all the hospitals, with the view of increasing the pecuniary support at present received. The institution of *The Hospitals Week* by the Hospital Sunday Fund has afforded them a much needed opportunity of going out, as it were, into the highways and thickets of this vast metropolis with the view of awakening the indifferent, the thoughtless, and the ignorant to the claims and necessities of the hospitals. This year a determined effort in the direction of extending the area from which donations and subscriptions are received by the hospitals, will be made. Eighteen meetings in the whole are to be held all over London in the fortnight preceding Hospital Sunday; and the enthusiastic and representative meeting of hospital managers held at the Mansion House early in the present year, should result in making every one of these meetings fruitful in benefits to the hospitals. Union is strength, and the united effort of all the existing supporters of the metropolitan hospitals, under the generalship of their managers, working with one common object to secure a great awakening of public feeling and interest through these meetings, cannot but result in increased funds being received, not only by the Central Fund, but by every individual charity. To secure this result each hospital must continue to cultivate the new ground which will be opened up by the numerous public meetings to be held in June next.

Lastly, there is a feeling amongst many wealthy personages, as well as amongst the large majority of the people, in favour of a movement on behalf of the hospitals to celebrate the Queen's Jubilee. The women of England have decided to have a Fund all to themselves. Are the men of this generation so careless, and is their loyalty so feeble, that they will rest content to sit still and do nothing to give expression to their sentiments? We do not believe it. We are sure that the feeling expressed by a little boy when he asked his father, "Mayn't I show my loyalty and give my penny to the Queen's Fund, father, because I am a boy and not a girl?" will yet find utterance in a combined effort to give practical expression to the loyal sentiments of the sterner sex. What happier channel could be selected for such a purpose than a fund in aid of all the hospitals, with the object of establishing the voluntary principle of hospital support upon a lasting basis?

Our readers will agree with us, that persistent effort, and not panic-stricken utterance, is the need of the hour. This being so, we support *The Lancet* in its appeal to the heads of the medical colleges and leading hospitals, in favour of steps being immediately taken to organise an effort to raise a Jubilee Hospital Fund.

